# Lyophilized Emulsions of Thymol and Eugenol Essential Oils Encapsulated in Cellulose

**DOI:** 10.3390/polym16101422

**Published:** 2024-05-17

**Authors:** Koranit Shlosman, Dmitry M. Rein, Rotem Shemesh, Yachin Cohen

**Affiliations:** 1 The Interdepartmental Program in Polymer Engineering, Technion-Israel Institute of Technology, Haifa 32000, Israel; skoranit@bazan.co.il; 2 R&D and Customer Service Department Carmel Olefins Ltd., Haifa 31014, Israel; srotem@bazan.co.il; 3 Department of Chemical Engineering, Technion-Israel Institute of Technology, Haifa 32000, Israel; cerycdr@technion.ac.il

**Keywords:** cellulose capsules, essential oils, lyophilization, volatiles release rate, biopolymer, bio-based material

## Abstract

Efforts to tap into the broad antimicrobial, insecticidal, and antioxidant activities of essential oils (EOs) are limited due to their strong odor and susceptibility to light and oxidation. Encapsulation of EOs and subsequent drying overcome these limitations and extend their applications. This study characterized freeze-dried (lyophilized) emulsions of eugenol (EU) and thymol (TY) EOs, encapsulated by chemically unmodified cellulose, a sustainable and low-cost resource. High-resolution scanning electron microscopy showed successful lyophilization. While the observed “flake-like” structure of the powders differed significantly from that of the emulsified microcapsules, useful properties were retained. Fourier transform infrared spectroscopy confirmed the presence of EOs in their corresponding powders and thermo-gravimetric analysis demonstrated high encapsulation efficiency (87–88%), improved thermal stability and resistance to evaporation, and slow EO release rates in comparison to their free forms. The lightweight and low-cost cellulose encapsulation, together with the results showing retained properties of the dried powder, enable the use of EOs in applications requiring high temperatures, such as EO incorporation into polymer films, that can be used to protect agricultural crops from microbial infections.

## 1. Introduction

Essential oils (EOs) are natural compounds present in aromatic and medicinal plants known for their antimicrobial and pesticidal activities and have been the subject of many studies [1,2,3,4,5,6,7,8,9,10]. EOs are safe for humans, animals, and the environment, and are used in many applications, including food, cosmetics, personal protective preparations, pharmaceutics, and agriculture [4,11,12,13]. For example, polymer nanofibers containing an inclusion complex of prochloraz in hydroxypropyl-γ-cyclodextrin was recently reported [14]. EOs are also very volatile, an advantage in applications requiring no direct contact between the target surface/microorganism and the EO-releasing device, for example, insect repellants for crop protection [4]. Yet, EOs are limited by vulnerability to light, high temperature, moisture, and oxygen [12,15,16], and their distinctive strong odor, and poor solubility in water [17,18]. These characteristics limit their use in industrial applications [19]. Encapsulation of EOs can overcome these barriers by decreasing the interactions between the “core” (i.e., encapsulated ingredient) and the environment [2,18,20,21,22]. It is an effective technique to decrease their volatility and sensitivity to environmental conditions, to enable their controlled release [2,23], decrease their degradation [1], mask their strong organoleptic characteristics [18], and enhance their antimicrobial activity [3,15,24].

Drying is a common and significant process to convert encapsulated particle emulsions into powders. It widens the application range of encapsulated EOs, increases their shelf life, and reduces the weight of the final product, resulting in a more economical transportation [12,25]. Spray-drying and freeze-drying (also referred to as lyophilization) are common drying methods used in the pharmaceutical and food industries. Spray-drying is the oldest and most robust method, and is industrially favorable as it enables production of large quantities at minimal costs [26]. However, the high temperatures used in the spray-drying process can damage the sensitive ingredients in the sample and may require a more complex geometry of the high-pressure spraying nozzle. The spray-drying process also involves high operation and installation costs [27]. Consequently, the lyophilization process, which is slower and entails higher energy consumption, is more suitable for delicate applications [28,29]. During lyophilization, low temperatures (below the freezing point of water) and a very low pressure (high vacuum) are maintained to enable the sublimation of water (ice) [22]. The lyophilization process is composed of mainly four stages: (1) freezing, (2) ice sublimation (primary drying), (3) desorption of unfrozen water (secondary drying), and (4) storage [26,30,31,32]. Lyophilization preserves natural ingredients by minimizing their deterioration due to oxidation [20] and maintains existing emulsion-like cell structures and characteristics of the original natural ingredients. In addition, due to the ice crystals formed during the freezing stage, lyophilization results in a porous structure, which may provide better control of the encapsulated materials’ release [33]. Lyophilization was shown to be a useful technique for the formation of cellulose aerogels from hydrogels [32]. It was shown to induce a microstructure with useful properties for advanced applications, such as water removal from waste emulsions, where the aerogel is actually re-introduced into a liquid environment [34,35].

Our previous work described a novel method to encapsulate thymol (TY) and eugenol (EU) EOs using chemically unmodified cellulose [36]. Regenerated cellulose hydrogel obtained from micro-crystalline cellulose (MCC) was used to form a continuous encapsulating shell around the EOs to form micro-particles with diameters of 1–5 µm. For EO encapsulation, a high-pressure homogenization process (HPH) was applied, while testing three pressures (5000, 10,000, and 20,000 PSI) and two cellulose:EO weight ratios (1:1 and 1:8). It was found that the pressure applied during the HPH process did not affect the capsule size, while the cellulose:EO ratio substantially affected both the capsules size and anti-mold activity of the emulsions. The 1:8 cellulose:EO ratio (optimally prepared by HPH at 10,000 PSI) yielded capsules of a diameter of about 5 µm that exhibited excellent anti-mold activity for both EU and TY in the alfalfa plant, used as a model system for hay.

To further study aqueous emulsions of cellulose-encapsulated EOs, it is of interest to investigate the structure, properties, and the possible applications of dried emulsions. While encapsulation can alleviate some limitations of EOs, the drying of the water-based emulsions may further contribute to their applicability in industrial uses. For example, their significantly lighter weight lowers transport costs and extends shelf life. Cellulose-encapsulated EO powders may be more stable in higher-temperature processes, such as compounding with polymers and film formation, thus presenting a significant bio-based alternative to the use of synthetic chemical pesticides. The current work aimed to characterize the structure and anti-mold capacities of freeze-dried TY and EU encapsulated by unmodified cellulose.

## 2. Materials and Methods

### 2.1. Materials

Eugenol (98%), thymol (98.5%), NaOH, and microcrystalline cellulose (MCC) powder (batch No. MKCJ3230, particle size 70–250 µm) were purchased from Sigma–Aldrich Co. (Rehovot, Israel). A similar MCC was previously characterized as having molecular weight of 50 kDa [37]. Deionized water was used to prepare the emulsion samples.

### 2.2. Methods

#### 2.2.1. Encapsulation of Essential Oil Emulsions and Their Lyophilization

EO–cellulose emulsions were prepared by homogenizing cellulose hydrogel suspension with EOs, as previously reported by Shlosman et al. [36]. Briefly, the hydrogel suspension was prepared by regeneration from a solution of MCC aqueous 7 wt.% NaOH at −17 °C. Emulsions were prepared at 1:8 cellulose: EO ratio was made by homogenizing a mixture consisting of 36.2% regenerated cellulose hydrogel (containing 2 gr of cellulose), 10.7% (16 gr) EO (EU or TY), and 53.1% water using a mechanical homogenizer (T18 digital Ultra-Turrax, IKA Works, Staufen, Germany), followed by high-pressure homogenization (HPH, Model LM-20 microfluidizer, Microfluidics, Newton, MA, USA) at 10,000 PSI. The content of emulsified medium EO in the emulsion corresponds to 88.9%. The obtained emulsions were centrifuged at a speed of 6000 rpm (relative centrifugal force about 2400× *g*) for 7 min in an MRC benchtop centrifuge (SCEN-206, MRC laboratory Instruments Ltd., Holon, Israel). Excess water and EOs were removed manually (by pipette) and the dense emulsion was then lyophilized.

Approximately 1.5 g centrifuged emulsions were placed in a 10 mL glass vial and positioned on the shelves of a Labconco FreeZone^®^ stoppering tray dryer (model 7948030, Labconco, Kansas, MI, USA). The lyophilization coil temperature was set to −50 °C and pressure was set to 0.2 mbar. The samples were cooled to −40 °C and maintained at this temperature for 48 h, after which, they were heated to room temperature and removed from the lyophilizer. For reference, a sample of cellulose hydrogel suspension without EOs underwent homogenizations, centrifugation, and lyophilization. The identification of the studied samples is given in Table 1.

#### 2.2.2. Characterization

##### Morphological Analysis via Scanning Electron Microscopy and Light Microscopy

The morphology of lyophilized emulsions was studied by scanning electron microscopy (SEM) and light microscopy. A Zeiss Ultra Plus high-resolution scanning electron microscope (Carl Zeiss, Jena, Germany) equipped with a Schottky field-emission gun was used. Specimens were placed on specimen holders and imaged at a low acceleration voltage of 1.0–1.1 kV without metal coating using Everhart–Thornley (“SE2”) and the In-the-column (InLens) secondary electron imaging detectors. Images were acquired using SmartSEM softwareV7 (Carl Zeiss, Jena, Germany) and analyzed by ImageJ 1.53 (U.S. National Institutes of Health, Bethesda, MD, USA). The overall structure of the lyophilized specimens and after slight milling (placed on a glass slide) was observed using an Olympus BX60 light microscope equipped with an MPLFLN lens unit, using a transmission light source (Evident Corp., Waltham, MA, USA). Stream Essentials 2.4 software (Olympus Scientific Solutions, Evident Corp., Waltham, MA, USA) was used to record and analyze images.

##### Chemical Composition of the Lyophilized Powders

Attenuated total reflectance Fourier transform infrared spectroscopy (ATR-FTIR) spectroscopy was used to identify the functional groups of EOs and cellulose in respective powders. A Perkin Elmer Fourier transform infrared spectrophotometer (Perkin Elmer FTIR Spectrum BX II, Waltham, MA, USA) was used in attenuated total reflectance (ATR) mode and acquired at a spectral range of 4000–500 cm^−1^ at a resolution of 4 cm^−1^, for 32 cycles. Perkin Elmer spectrum IR version 10.6.1 software was used for the analysis.

##### Thermal Analysis

The thermal stability and composition of the micro-capsules, as well as the EO releasing rates from the capsules, were studied using thermo-gravimetric analysis (TGA) (TGA Q5000 system, TA instruments, New Castle, DE, USA) equipped with a ceramic pan. Dynamic TGA was performed to assess thermal stability and % encapsulation efficiency (%EE), and for compositional analysis. Samples were heated from room temperature up to 800 °C at a heating rate of 10 °C/min, under nitrogen atmosphere. To study the EO release rates from the capsules, a static TGA program was used: the samples were heated to 40 °C and 50 °C (for EU and TY, respectively) at a heating rate of 20 °C/min and maintained at this temperature for 900 min. The weight loss in both dynamic and static TGA modes was monitored throughout the experiment and EO content in each sample was calculated. Each measurement point represents an average of two measurements and a bar representing the upper and lower values is specified in the corresponding graphs.

Encapsulation efficiency: Different procedures have been reported in the literature for calculation of the % encapsulation efficiency (EE), most of which are based on different extraction and filtration methods [26,38,39,40,41]. All the methods require knowledge of the initial amount of EO incorporated into the formula and the actual amount of EO present in the sample, as given by Equation (1) [39]:

(1) $$EE\left(\%\right)=\frac{EO\;content\;in\;powder}{EO\;content\;in\;feed\;liquid}\times 100\%$$

## 3. Results and Discussion

### 3.1. Morphology of Lyophilized Emulsions

**Figure 1:**
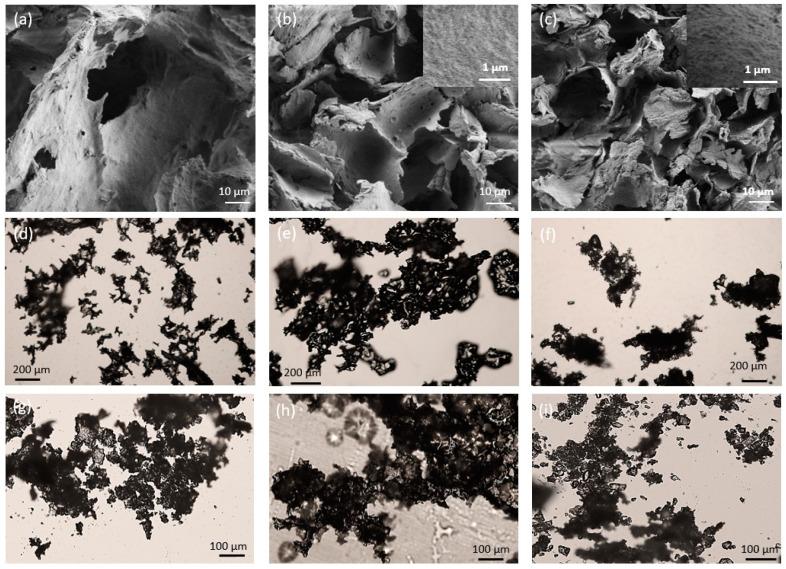
SEM (**a**–**c**) and light microscope (**d**–**i**) images of lyophilized samples of (**a**,**d**,**g**) hydrogel_lyo, (**b**,**e**,**h**) EU-1:8-10k_lyo and (**c**,**f**,**i**) TY-1:8-10k_lyo. Lyophilized specimens (**d**–**f**) before and (**g**–**i**) after light milling.

Figure 1a–c depicts the morphology of the lyophilized emulsions, as viewed by SEM. A morphology of irregular porous flakes with sharp edges was observed and is considered to be made of the aggregated particles. In contrast to the emulsions [36], the cryo-electron microscopy found no individual micron-sized particles. A similar morphology of freeze dried capsules (not ground) was previously reported by several researchers, who described the structure as “cake-like” [26], “slab-like” [42], “flake-like” [43], and “broken glass-like” [28,39], implying that lyophilized material is characterized by non-spherical particles. This structure likely resulted from the concentration of emulsion droplets and remaining free hydrogel particles, first by centrifugation, followed by the front of crystallizing water during freezing. During coalescence and vitrification at low temperatures, the forces between the impinging crystal fronts are not strong enough to break the vitrified mass into droplets. The pores may have formed by the sublimation of ice crystals [26,28,30,39,42,43]. Agglomeration of cellulose fibrils into sheet-like structures, induced by the ice crystal formation, has been observed in freeze-dried bacterial cellulose (BC) cryogels [32]. Freeze-dried aqueous BC suspensions were shown to exhibit “interlinked sheets” with “irregularly shaped macro-pores” [35].

SEM analyses performed at higher magnification are presented in Figures S1–S3 in the Supplementary Materials. The higher resolution enabled imaging the hydrogel structure as a porous network, similar to that observed by cryogenic SEM analysis of the original emulsions [36]. Flake thickness was found to be between 80 and 300 nm.

Light microscopy images of the lyophilized powder structure before (Figure 1d–f) and after light manual milling (Figure 1g–i) showed irregular-shaped agglomerates with a flaky texture, regardless of the application of milling. Light manual milling had no effect on the observed particles’ morphology. Thus, it was concluded that SEM images captured directly after sample lyophilization well represent the actual sample morphology.

### 3.2. Chemical Composition of the Lyophilized Powders Attenuated Total Reflectance Fourier Transform Infrared Spectroscopy (ATR-FTIR)

**Figure 2:**
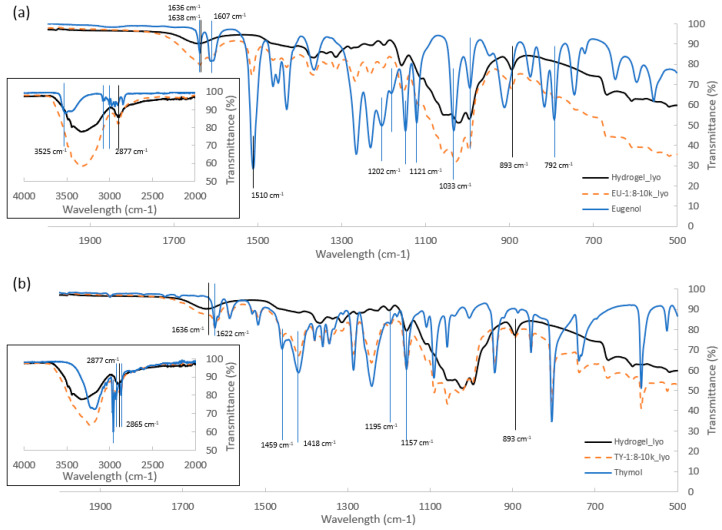
FTIR-ATR spectra of Hydrogel_lyo together with (**a**) free EU and EU-1:8-10k_lyo and (**b**) free TY and TY-1:8-10k_lyo.

ATR-FTIR spectra of the lyophilized samples of cellulose hydrogel, cellulose-encapsulated EO and TY, and free EU and TY, are presented in Figure 2. Absorption peaks which are characteristic of cellulose, EU, and TY [36] were observed, and indicated successful encapsulation of EU and TY. The characteristic bands of cellulose attributed to the presence of OH groups (3000–3700 cm^−1^) and C–H stretching vibration (893 cm^−1^, 2877 cm^−1^ and 1030 cm^−1^) were clearly seen in the spectra of lyophilized cellulose. The O–H bending vibration (1636 cm^−1^), due to adsorbed water molecules, was also observed in the lyophilized cellulose FTIR-ATR spectra [44,45]. Characteristic EU FTIR bands included stretching vibration of the aromatic C–C bond (1510, 1607, 1638, and 3525 cm^−1^), asymmetric stretch of C–O–C bonds (1121, 1147, 1181, and 1202 cm^−1^), alcoholic C–O vibrations (1033 cm^−1^), and C–H bonding vibrations originated in the CH_2_ and CH_3_ groups (792, 994, 3000, and 3062 cm^−1^) [24,36,46,47,48,49]. TY FTIR was characterized by phenolic O–H stretching vibration (3000–3500 cm^−1^), C–O stretching vibration (1157, 1195 cm^−1^), C–H bonds (2865, 2926, 2958 cm^−1^), C–H stretching vibrations (1418, 1459 cm^−1^), and aromatic C=C stretching of benzene ring (1622 cm^−1^) bands [36,50,51,52]. Similar bands were also observed in the corresponding lyophilized samples EU-1:8-10k_lyo and TY-1:8-10k_lyo indicating the presence of the respective EO in the lyophilized samples. Slight shifts of absorption bands were also observed, indicating different interactions between constituents [24]. The presence of cellulose and absorbed water in both lyophilized samples was also apparent from the wide band at 2980–3700 cm^−1^. Overall, FTIR analysis indicated successful lyophilization, whereby the EOs content was preserved in the capsules, in agreement with FTIR results previously reported for EO emulsions [36], which showed that no chemical changes occurred during encapsulation of EU and TY EOs.

### 3.3. Thermal Stability and EO Content of the Lyophilized Powders

**Figure 3:**
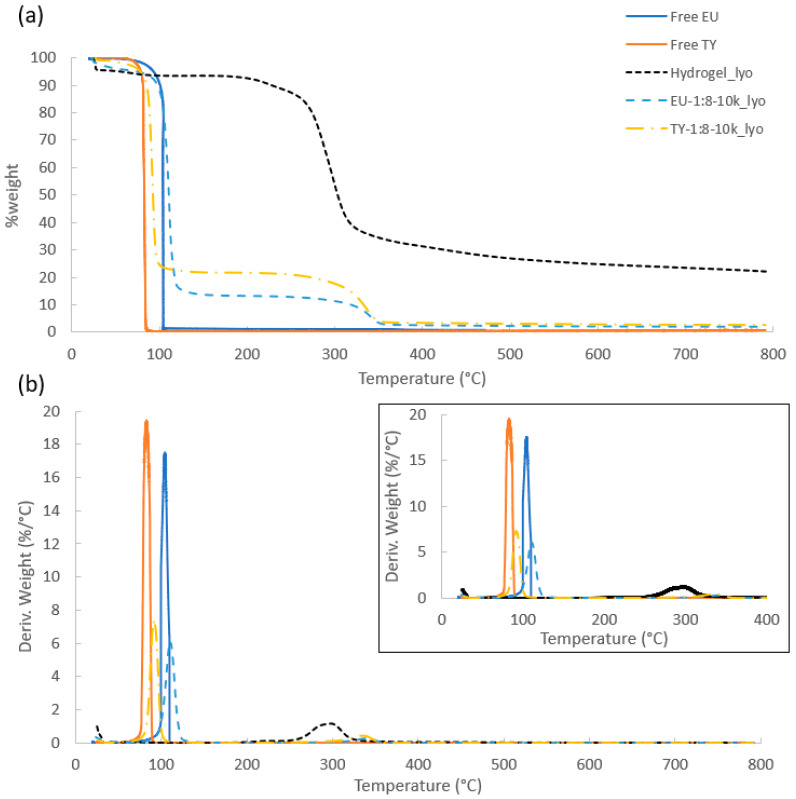
Thermal analyses of Hydrogel_lyo, free EU, free TY, EU-1:8-10k_lyo and TY-1:8-10k_lyo: (**a**) TGA and (**b**) DTG thermographs Inset: zoom-in of DTG in the range of 25–400 °C.

Thermo-gravimetric analysis (TGA) is a powerful tool that can be used to obtain information on the thermal stability, composition (including moisture content), % encapsulation efficiency (%EE) and release rates of encapsulated EOs after lyophilization [50,53,54]. TGA graphs taken in dynamic mode reflect the sample’s weight loss during its heating at a constant rate. Figure 3 presents TGA and derivative TG (DTG) of Hydrogel_lyo, free EU and TY, EU-1:8-10k_lyo and TY-1:8-10k_lyo. The thermograms of free EU [24] and TY [52] are characterized by a single peak in the temperature range of 25–100 °C, attributed to their evaporation, due to their high volatility. The differential analysis indicated that the peak temperatures of maximum rate for this phase transition were 104.6 and 84.2 °C for free EU and TY, respectively. The thermogram of Hydrogel_lyo was characterized by two weight loss stages. The first, in the temperature range of 25–100 °C, can be attributed to the loss of surface-absorbed water [55,56] and corresponded to 7.6% of the sample weight. The second weight loss stage of about 70%, seen in the temperature range of 100–700 °C, can be attributed to cellulose decomposition, by the breakdown of glycosyl units. A 21.6% residue (quantified at 800 °C) was present in the sample. The thermograph was slightly different than that of MCC thermograms published in the literature, due to the more amorphous structure and lower molecular weight of the cellulose hydrogel compared to crystalline MCC [56,57], as was shown in several studies characterizing regenerated cellulose by viscosity and by GPC [58], and its structure by X-ray diffraction [59,60]. The thermograms of EU-1:8-10k_lyo and TY-1:8-10k_lyo were characterized by three weight loss stages. The first, attributed to moisture contents of 5.2% and 1%, was recorded in the temperature range of 25–65 °C and 25–50 °C for EU-1:8-10k_lyo and TY-1:8-10k_lyo, respectively. The second weight loss, attributed to the evaporation of the encapsulated EO, measured 77.5% and 78.5% for EU-1:8-10k_lyo and TY-1:8-10k_lyo, respectively. This quantification was performed up to the temperature of 200 °C, starting at the temperature at which water ceased to evaporate. The third decomposition stage, observed in the temperature range of 200–500 °C, can be attributed to the decomposition of cellulose, and measured 13.9% and 16.7% for EU-1:8-10k_lyo and TY-1:8-10k_lyo, respectively. The residual content, quantified at 800 °C, measured 2.5% and 3.5% for EU-1:8-10k_lyo and TY-1:8-10k_lyo, respectively. These results complement the FTIR analysis and indicate that EU and TY were well encapsulated by the regenerated cellulose hydrogel. When considering the temperature at which the EOs begin to evaporate and the peak temperature of maximum evaporation, it can be seen that the peak temperature at the maximum evaporation rate increased from 104.6 °C to 110 °C and from 82.4 °C to 91.7 °C for EU-1:8-10k_lyo and TY-1:8-10k_lyo, respectively. These results support the suggested use of the dried capsules in applications demanding high temperature, such as compounding and extrusion with polymers, used in packaging [61,62] and post-harvest disease protection [63]. An interesting effect was observed regarding the maximum evaporation rate temperature for the decomposition of cellulose: while the peak temperature for Hydrogel_lyo was 303 °C, when used as a shell material, the peak temperature increased to about 340 °C for both encapsulated EO samples. This can be explained by interactions between cellulose and EOs, leading to a more thermally stable structure. The weight percentages of each ingredient in the different samples and peak temperature of EO evaporation are summarized in Table 2.

By using Equation (1), a very high %EE of 87% and 88% was achieved for EU-1:8-10k_lyo and TY-1:8-10k_lyo, respectively. While high %EE can also be achieved using spray-drying, the high %EE of lyophilization with the use of crop-waste encapsulating material can provide effective alternative for industrial-scale processes [64,65,66].

The thermal analyses complement the FTIR tests, demonstrating the successful inclusions of EOs in an unmodified cellulose shell, and their retention in the lyophilized structure. Increased thermal stability observed for encapsulated EU and TY compared to their corresponding free EOs, indicates that their encapsulation increases their resistance to evaporation [41,50,52,67,68].

**Figure 4:**
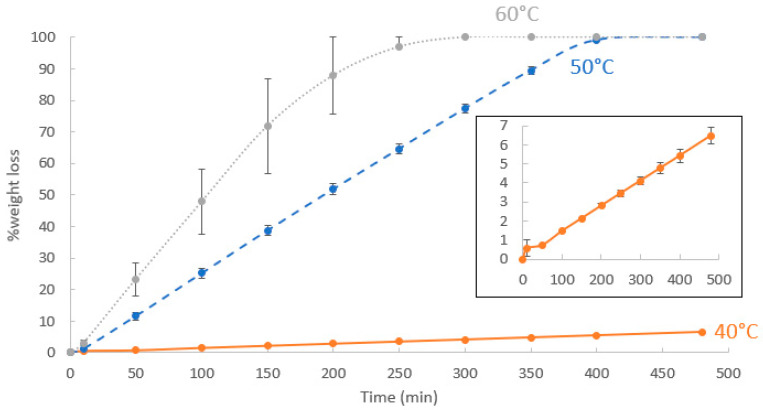
Static TGA of free TY performed at 40, 50 and 60 °C. Each point represents an average of two measurements; the error bars represent the upper and lower values. Inset—zoom-in of the measurements at 40 °C.

Release rate: Static TGA can also provide valuable information on EO release rates from the capsules. In such assessments, the samples were heated to a desired temperature (40 °C for EU and EU-1:8-10k_lyo and 50 °C for TY and TY-1:8-10k_lyo) and retained at that temperature for 900 min, while the weight loss was monitored. In order to optimize the test temperature for the different samples, a preliminary test was performed on free TY at 40, 50 and 60 °C, as shown in Figure 4. It can be seen that while maintained at 60 °C, all the TY evaporated within 300 min, while at 40 °C, there was almost no loss of TY (6.1% weight loss after 480 min), attributed to the fact that TY is solid at this temperature (its melting temperature is 52 °C [51]), with low vapor pressure. Thus, the temperature for the experiment testing TY release was set to 50 °C. Unlike TY, EU is liquid at room temperature, enabling the above test to be performed at 40 °C in an acceptable time frame; hydrogel_lyo was also analyzed at 40 °C.

**Figure 5:**
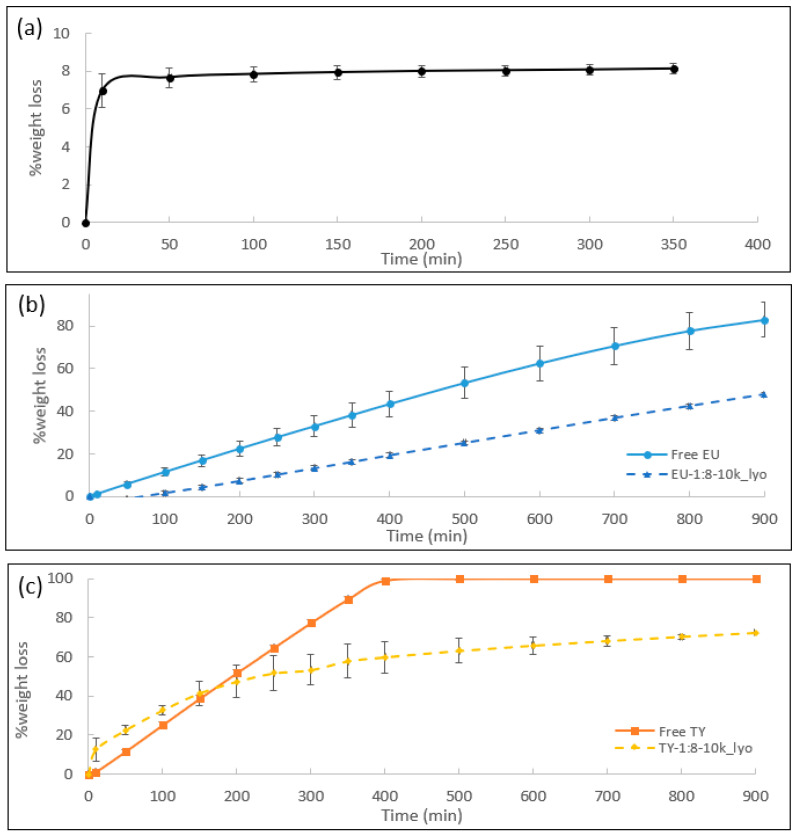
Static TGA; % weight loss over time for (**a**) Hydrogel_lyo performed at 40 °C, (**b**) free EU and EU-1:8-10k_lyo performed at 40 °C, and (**c**) free TY and TY-1:8-10k_lyo performed at 50 °C. An average of two measurements is presented. The error bars connect the measured values, when shown, otherwise the measurements were identical. In (**b**,**c**), for comparison purpose with free EO, the weight loss of lyophilized samples is presented after water subtraction and normalization to EO content, as explained in the text and SI.

Figure 5a–c shows the static TGA measurements of the studied samples. Figure 5a depicts the weight loss % of Hydrogel_lyo at 40 °C. Neither thermal degradation nor evaporation occurred at this temperature, therefore the weight loss observed can be fully attributed to moisture absorbed on the cellulose surface (corresponding to the amount observed in Figure 3 and listed in Table 2). After 10 min, 50 min, and 100 min, 76, 90, and 94% of the moisture content had evaporated, respectively, and a plateau was reached within 150 min. For the lyophilized samples, the initial weight loss (during the first 150 min) includes both water and EO evaporation. Therefore, in this time frame, the weight loss of water was considered to be equal to the water weight loss % observed in the Hydrogel_Lyo, Figure 5a (raw data and water-subtracted data are given in Tables S1 and S2, respectively). The water-subtracted weight loss of lyophilized samples was further normalized by dividing with the EO content of the respective lyophilized powders and presented in Figure 5b,c (normalized data are given in Table S3 in SI).

The main quantitative analysis was made within a time frame of 400 min, but to further understand the release rates at much longer times, weight loss was monitored for 900 min at the predefined temperature. Free EU and EU-1:8-10k_lyo exhibited a linear rate of weight loss (R^2^ = 0.99, slope = 0.1%/min and R^2^ = 0.99, slope = 0.05%/min, respectively). Weight loss of free EU measured 43% after 400 min and 83% after 900 min, while EU-1:8-10k_lyo exhibited only 19% weight loss after 400 min and 48% weight loss after 900 min. Taken together, the encapsulated EU exhibited a significantly lower weight loss rate than free EU (by about 45% after 400 and by 58% after 900 min). Free TY also exhibited a linear rate of weight loss (R^2^ = 0.99, slope = 0.3%/min), with 99% of the total weight lost within 400 min. Unlike neat TY, TY-1:8-10k_lyo presented a non-linear release rate, with two successive nearly linear release processes: first, a quick release stage within a time interval of 0–150 min (R^2^ = 0.93, slope = 0.2%/min) with a total weight loss of 33%, and a second stage, characterized by a much slower release rate after 200 min (R^2^ = 0.95, slope = 0.03%/min), ending with a weight loss of 57% after 900 min.

Such forms of weight loss and the difference between the release profiles of the encapsulated EU and TY can be explained by consideration of the components’ thermodynamic properties and the capsule structure. The higher boiling point of EU compared to TY (254 °C and 233 °C, respectively) [69,70] implies that TY possess a higher vapor pressure in the liquid form in comparison to EU. Thus, the initial release rate TY is higher than that of EU at the tested temperatures (50 °C and 40 °C, respectively). Additionally, the encapsulating shells of the initial emulsions present another barrier for EO diffusion. Owing to breakage of a certain portion of the capsules during lyophilization, free EO can be considered to be present in the lyophilized powder in addition to encapsulated EO, which affects the release profile. The static TGA results complement thermal analysis performed by dynamic TGA and further demonstrate the successful encapsulation of EOs in the powder form. The release rate of EOs is a function of their volatility (i.e., vapor pressure); by increasing EO resistance to evaporation by their encapsulation (as shown in Figure 3), their release rate is slowed as EO diffusion through the capsule shell is slower than its diffusion in the free form. Such decreased release rates improve EO retention, which is a particular benefit for certain applications, e.g., incorporation of EO-loaded capsules into polymeric materials for enhanced antimicrobial activity of plastic films.

## 4. Conclusions

In this work, emulsions of EU and TY encapsulated in cellulose were successfully dried by lyophilization. The combination of the previously reported EO encapsulation method, with a subsequent lyophilization process resulted in high encapsulation efficiency and fabrication of well-dried emulsions, with extremely low moisture content, as well as maintenance of the porous structure. The lyophilized powder exhibits a “flake-like” structure, significantly different from that of the emulsified microcapsules. The chemically unmodified cellulose shell material increased thermal stability and decreased the release of the encapsulated EOs. When compared to the liquid emulsions, the dry form of encapsulated EOs extends potential industrial applications, such as EO incorporation into products that are subjected to intense thermal treatment, including compounding with polymer melts and fabrication of plastic articles. This can provide prolonged antimicrobial activity in household and agricultural applications with a reduced harmful impact on health and the environment.

## Figures and Tables

**Table 1:** Tested capsules.

Sample ID	EO ^1^ Type
Hydrogel_lyo	None
EU-1:8-10k_lyo	EU
TY-1:8-10k_lyo	TY

^1^ EO—essential oil.

**Table 2:** Summary of compositions and thermal stability of tested samples ^1^.

Sample	Moisture Content (%)	EO Content (%)	Decomposed Cellulose Content (%)	Peak Temp. of EO Maximum Evaporation Rate (°C)
Hydrogel_lyo	7.6 (2.3)	0	70 (0.1)	-
Free EU	0	100	0	104.6
Free TY	0	100	0	82.4
EU-1:8-10k_lyo	5.2 (1.7)	77.5 (9.4)	13.9 (3.8)	110
TY-1:8-10k_lyo	1 (0.8)	78.5 (3.2)	16.7 (5.9)	91.7

^1^ Average of two measurements (difference between the two measurements).

## Data Availability

The original contributions presented in the study are included in the article and Supplementary Materials, further inquiries can be directed to the corresponding author.

## Supplementary Materials

The following supporting information can be downloaded at: https://www.mdpi.com/article/10.3390/polym16101422/s1, Figure S1: HR-SEM micrographs of hydrogel_lyo at different magnifications. Dashed yellow circles indicate the area focused in the subsequent image; Figure S2: HR-SEM micrographs of EU-1:8-10k_lyo at different resolutions magnifications. Dashed yellow circles indicate the area focused in the subsequent image; Figure S3: HR-SEM micrographs of TY-1:8-10k_lyo at different resolutions magnifications. Dashed yellow circles indicate the area focused in the subsequent image; Table S1: TGA Raw data for weight loss; Table S2: TGA water-reduced data for weight loss: Eugenol @ 40 C, Thymol @ 50 C; Table S3: TGA normalized data for weight loss.
