# Supplementary material for: Lyophilized Emulsions of Thymol and Eugenol Essential Oils Encapsulated in Cellulose
Source: Polymers (Basel). 2024 May 17;16(10):1422. doi: 10.3390/polym16101422 (PMC11125086; doi:10.3390/polym16101422)
Supplement: Supplementary file 1 [file polymers-16-01422-s001.zip › polymers-2993629-supplementary.pdf]

Supporting information

Lyophilized Emulsions of Thymol and Eugenol Essential Oils Encapsulated by Cellulose

Koranit Shlosman[1,2], Dmitry M. Rein[3], Rotem Shemesh[2], Yachin Cohen[3,+]

1 The Interdepartmental program in polymer engineering, Technion-Israel Institute of Technology, 32000 Haifa, Israel

2 Carmel Olefins Ltd., 31014 Haifa, Israel

3 Faculty of Chemical Engineering, Technion-Israel Institute of Technology, 32000 Haifa, Israel

+ Corresponding author, email: yachinc@technion.ac.il

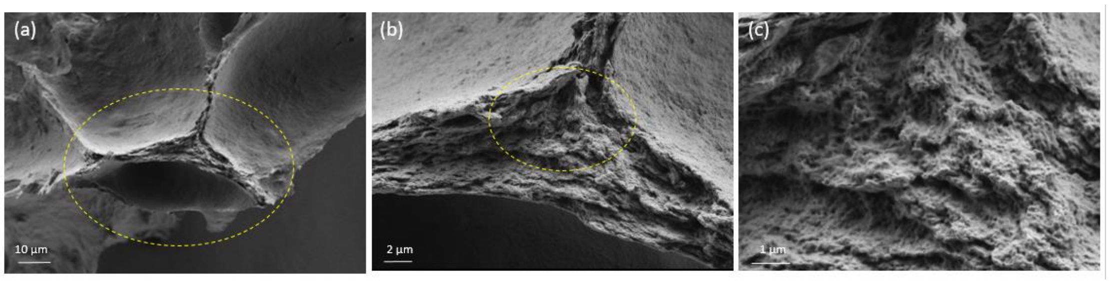

Figure S1: HR-SEM micrographs of hydrogel_lyo at different magnifications. Dashed yellow circles indicate the area focused in the subsequent image.

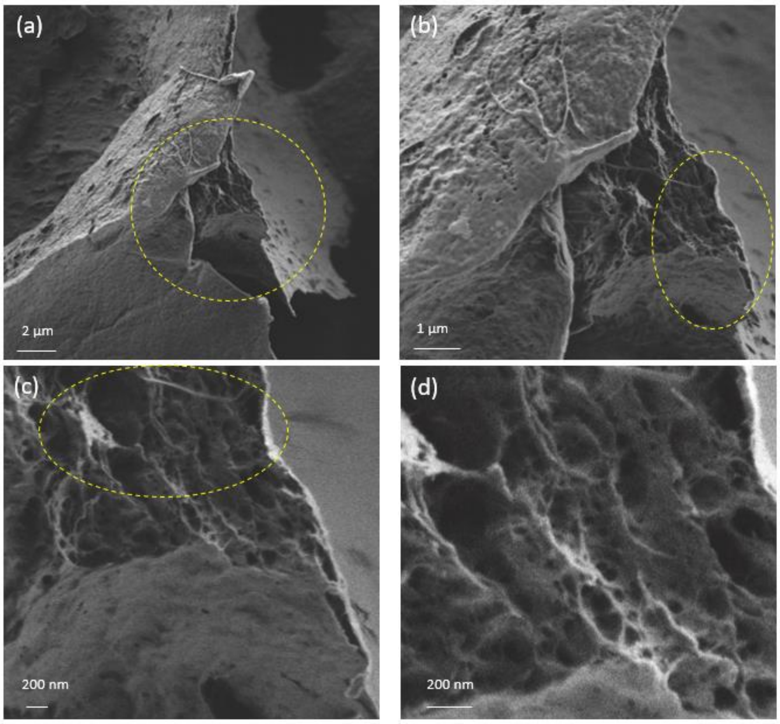

Figure S2: HR-SEM micrographs of EU-1:8-10k_lyo at different resolutions magnifications. Dashed yellow circles indicate the area focused in the subsequent image.

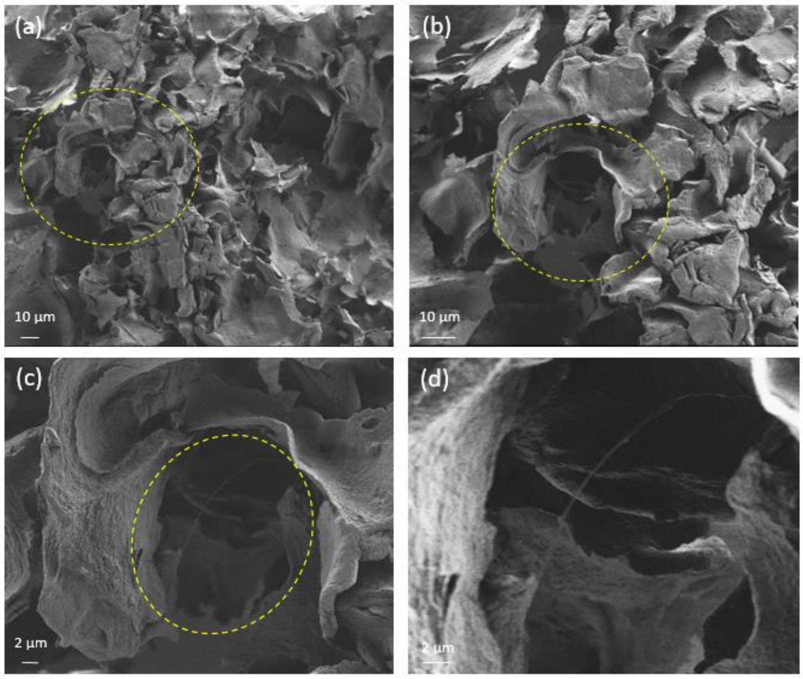

Figure S3: HR-SEM micrographs of TY-1:8-10k_lyo at different resolutions magnifications. Dashed yellow circles indicate the area focused in the subsequent image.

Table S1: TGA Raw data for weight loss

Weight loss (%)	Time (min)														
	0	10	50	100	150	200	250	300	350	400	500	600	700	800	900
Hydrogel_lyo	0	7.0	7.7	7.8	7.9	8.0	8.0	8.1	8.1						
neat TY	0	1.2	11.6	25.2	38.7	51.8	64.6	77.3	89.4	99.0	100.0	100	100	100	100
TY-1:8-10k_lyo	0	10.7	18.6	26.7	33.6	38.2	41.6	42.9	46.4	48.0	50.5	52.6	54.5	56.2	57.7
neat EU	0	1.1	5.8	11.4	16.9	22.4	27.8	33.0	38.2	43.4	53.3	62.4	70.6	77.7	82.9
EU-1:8-10k_lyo	0	1.7	3.9	6.2	8.5	10.9	13.1	15.5	17.8	20.2	24.7	29.2	33.74	38.07	42.3

Table S2: TGA water-reduced data for weight loss: Eugenol @ 40 C, Thymol @ 50 C

Weight loss (%)	Time (min)														
	0	10	50	100	150	200	250	300	350	400	500	600	700	800	900
neat TY	0	1.2	11.6	25.2	38.7	51.8	64.6	77.3	89.4	99.0	100.0	100.0	100.0	100.0	100.0
TY-1:8-10k_lyo	0	9.9	17.7	25.7	32.6	37.2	40.6	41.9	45.4	47.0	49.5	51.6	53.5	55.2	56.7
neat EU	0	1.1	5.8	11.4	16.9	22.4	27.8	33.0	38.2	43.4	53.3	62.4	70.6	77.7	82.9
EU-1:8-10k_lyo	0	-2.2	-0.8	1.3	3.3	5.7	7.9	10.3	12.6	15.0	19.5	24.0	28.5	32.9	37.1

Equation S1: $TY\ water - reduced\ data_{time\ t} = \%\ weight\ loss_{time\ t,\mathrm{TY-1:8-10k_{lyo}}} - \frac{\%\ weight\ loss_{time\ t,Hydrogel_{Lyo}}}{8.1} * 10\%$

Equation S2: $EU\ \ water - reduced\ data_{time\ t} = \%\ weight\ loss_{time\ t,\mathrm{EU-1:8-10k_{lyo}}} - \frac{\%\ weight\ loss_{time\ t,Hydrogel_{Lyo}}}{8.1} * 5.2\%$

Table S3: TGA normalized data for weight loss.

Weight loss (%)	Time (min)														
	0	10	50	100	150	200	250	300	350	400	500	600	700	800	900
Free TY	0.0	1.2	11.6	25.2	38.7	51.8	64.6	77.3	89.4	99.0	100.0	100.0	100.0	100.0	100.0
TY-1:8-10k_lyo	0.0	12.6	22.6	32.8	41.5	47.4	51.7	53.3	57.9	59.8	63.1	65.8	68.1	70.3	72.3
Free EU	0.0	1.1	5.8	11.4	16.9	22.4	27.8	33.0	38.2	43.4	53.3	62.4	70.6	77.7	82.9
EU-1:8-10k_lyo	0.0	-2.9	-1.0	1.7	4.3	7.3	10.2	13.3	16.2	19.3	25.1	31.0	36.8	42.4	47.9

Equation S3: $TY\ normalized_{time\ t} = \frac{TY\ water-reduced\ data_{time\ t}}{0.785}$

Equation S4: $EU = \frac{EU\ water-reduced\ data_{time\ t}}{0.775}$
